# Proteogenomic Gene Structure Validation in the Pineapple Genome

**DOI:** 10.1021/acs.jproteome.3c00675

**Published:** 2024-04-23

**Authors:** Norazrin Ariffin, David Wells Newman, Michael G. Nelson, Ronan O’cualain, Simon J. Hubbard

**Affiliations:** † School of Biological Sciences, Faculty of Biology Medicine and Health, MAHSC, University of Manchester, Michael Smith Building, Oxford Road, Manchester M13 9PT, United Kingdom; ‡ Department of Agriculture Technology, Faculty of Agriculture, Universiti Putra Malaysia, Serdang 43400, Selangor Darul Ehsan, Malaysia

**Keywords:** proteomics, genomics, proteogenomics, computational biology, genome annotation

## Abstract

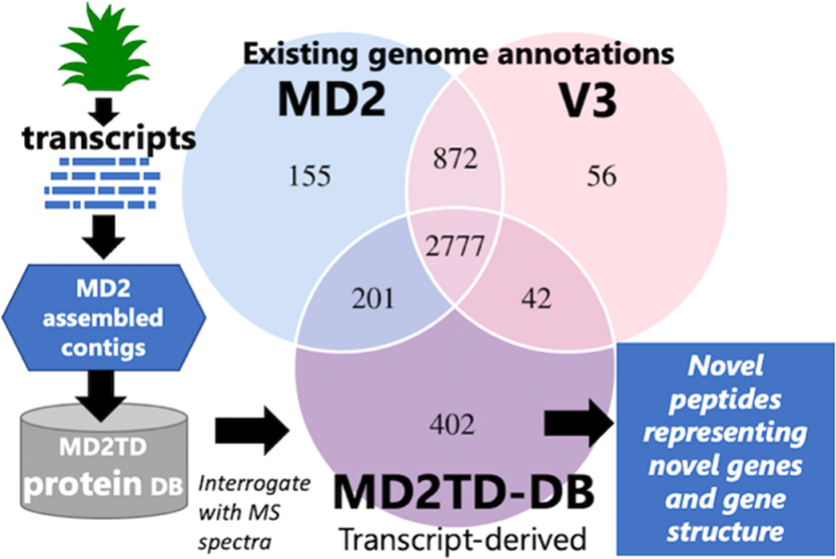

MD2 pineapple (*Ananas comosus*) is the second most important tropical crop that preserves crassulacean acid metabolism (CAM), which has high water-use efficiency and is fast becoming the most consumed fresh fruit worldwide. Despite the significance of environmental efficiency and popularity, until very recently, its genome sequence has not been determined and a high-quality annotated proteome has not been available. Here, we have undertaken a pilot proteogenomic study, analyzing the proteome of MD2 pineapple leaves using liquid chromatography-mass spectrometry (LC–MS/MS), which validates 1781 predicted proteins in the annotated F153 (V3) genome. In addition, a further 603 peptide identifications are found that map exclusively to an independent MD2 transcriptome-derived database but are not found in the standard F153 (V3) annotated proteome. Peptide identifications derived from these MD2 transcripts are also cross-referenced to a more recent and complete MD2 genome annotation, resulting in 402 nonoverlapping peptides, which in turn support 30 high-quality gene candidates novel to both pineapple genomes. Many of the validated F153 (V3) genes are also supported by an independent proteomics data set collected for an ornamental pineapple variety. The contigs and peptides have been mapped to the current F153 genome build and are available as bed files to display a custom gene track on the Ensembl Plants region viewer. These analyses add to the knowledge of experimentally validated pineapple genes and demonstrate the utility of transcript-derived proteomics to discover both novel genes and genetic structure in a plant genome, adding value to its annotation.

## Introduction

MD2 pineapple is a tropical fruit that originated from cross-breeding two pineapple hybrid strains, carried out by the Pineapple Research Institute, Hawaii, with a complex pedigree involving five generations of hybridization. The Pineapple plant is a diploid with 2*n* = 50 and a genome size of approximately 526Mb.^1^ Pineapple is propagated vegetatively, and this has led to increased heterozygosity in the population, adding additional complexity in the sequencing and deciphering of its genome. Ideally, a sequenced genome should capture a comprehensive set of predicted gene sequences, free from errors, and complete with annotated expression patterns and functional annotations. However, much of the genome annotation currently available is generated primarily from computational methods and hence would benefit from additional experimental support.

Genome annotations can be assisted by transcriptional and translational evidence that supports the predicted gene structure at a given genomic locus. For example, RNA sequencing can generate extensive transcriptome data^2^ in the form of cDNAs or expressed sequence tags (ESTs), which provide direct evidence of expression in the cells or tissues used. These resources can in turn be used in proteomics, where transcriptome data can be translated, with a program, such as Transdecoder,^3^ into meaningful protein sequences that include splice variants and alternate gene boundaries for their parental genes.^4,5^ Crucially, such transcriptome-informed proteomic experiments, when carefully controlled for false discoveries, can be highly informative of genes and gene structures that have been otherwise missed by the genome annotators. This technique, often referred to as proteogenomics, can be integrated into a proteomics pipeline to validate predicted genes and assist the discovery of potentially novel ones.^6,7^ By providing direct experimental evidence of gene expression at the protein level, proteogenomics has been applied in many organisms from human,^8^ bacteria and archaea,^9^ as well as several plant species, such as grapevine^10^ and rice.^11^ Proteogenomics is not only useful in detecting novel genes but also plays an important role in improving the established genome annotation for all organisms by the discovery of refined gene structures and gene boundaries.^12,13^ The combination of both transcriptomic and proteomic data can yield as much as a 60% improvement in the discovery of gene structure compared to the previous genome annotations, which is considered to be a major progression.^12,13^ Proteogenomics has even demonstrated its benefits in difficult systems with high GC content and divergence from known prokaryote gene models, identifying 41 novel protein-coding genes and refining 79 gene models in H37Rv strain of*Mycobacterium tuberculosis*.^14^

The work described here investigates the use of proteogenomics applications to improve pineapple genome annotations by using novel experimental data to refine existing genome annotations. Here, we initially used two previous pineapple genome sequences, generated by Ming et al.^15^ and Redwan et al.^16^ The first of these used both long and short read sequencing to sequence the pineapple based on a cross between the F153 variety and MD2 variety that was backcrossed to a wild pineapple relative, *Ananas bracteatus* accession CB5.^15^ Based on the assembly and use of three strains, we refer to this version from Ming and colleagues^15^ as the V3 genome. Subsequently, an independent study led by Redwan and colleagues added long-read sequencing to refine a draft of the MD2 genome, with 99.6% genome coverage with 27,017 predicted protein-coding genes.^16^ Hence, the latter MD2 genome can also be considered as a validation set for novel findings discovered with respect to the V3 genome, which were subsequently reported in the MD2 version. We have used protein annotations from this long-read, updated MD2 version here for the purposes of validating proteogenomics approaches.

Here, samples were generated from MD2 leaves to generate transcriptomes and protein samples. A subsequent *de novo* assembly of the MD2 pineapple transcriptome generated a contig set (TDMD2) that was used to predict protein sequences prior to proteogenomics analysis. Confident peptide identifications and their parent proteins/transcripts were compared to the genomes and their annotations, providing experimental validation for over 1800 MD2 genes, as well as paired contig/MS evidence for 30 novel candidate genes. Additionally, we mapped contigs containing novel peptides exclusive to TDMD2 contigs to a more recent F153 genome annotation available via Ensembl Plants, supporting visualization of the MD2 contigs and peptides as a custom Genes Track. Finally, a related ornamental pineapple genome has recently been sequenced and made available,^17^ and we compared an independent proteomics study^18^ from this leaf-chimeric red pineapple (*Ananas comosus* var. *bracteatus* f. *tricolor*) to our protein data. The results show the value of proteogenomics in generating experimental support for predicted proteins and genes and highlight the potential to find unannotated genes or novel gene structures in emerging genome annotations and specifically for the pineapple.

## Materials and Methods

### Sample Preparation and Proteolysis

The MD2 pineapple leaves were collected from ten distinct plants as one replicate, and three biological replicates were designed for RNA and protein extractions. The MD2 pineapple samples were collected at Ladang KOSAS, Banting Selangor, Malaysia. On picking, samples were wrapped in aluminum foil, labeled, and immediately kept in liquid nitrogen.

### MD2 Pineapple’s RNA Extraction

Approximately 100 mg of MD2 pineapple leaves was cleaned and ground into fine powder, and the total RNA was isolated using the PureLink Plant RNA Reagent (Invitrogen) according to the manufacturer’s protocol. RNA integrity number (RIN) with a value of 8.0 has been confirmed using a 2100 Bioanalyzer (Agilent Technologies) and prepared using Illumina’s kit following the protocol provided by the company. The sample has been processed to remove noncoding RNAs (such as rRNAs) before sequencing. The sequencing follows the manufacturer’s protocol for paired-end reads.

### Library Preparation and Sequencing

Preparation of the cDNA libraries was made according to the manufacturer’s instructions (Illumina, San Diego, CA). A total of 40 mg of RNA was purified using Sera-mag Magnetic Oligo (dT) Beads (Illumina) and eluted with 10 mM Tris-HCl. Next, the mRNA was fragmented using RNA Fragmentation Reagents (Ambion, Austin, TX) prior to cDNA synthesis. The fragmented mRNA was converted to double-stranded cDNA using a SuperScript Double-Stranded cDNA Synthesis kit (Invitrogen, Camarillo, CA) with random hexamer primers (Illumina). cDNA was proceeded to the next purification by using a QiaQuick PCR Purification Kit (Qiagen, Valencia, CA) followed by end-repair and phosphorylation by T4 DNA, Klenow DNA polymerases, and T4 PHK (NEB, Ipswich, MA). To create cDNA fragments with a single “A” base overhang at the 39th position end ready for subsequent Illumina paired-end adapter ligation, the 39 base pair fragments were adenylated using Klenow Exo- (Illumina). cDNA was excised from 2% TAE agarose gel and was later purified using a QIAquick Gel Extraction Kit (Qiagen). PCR Primer PE 1.0 and PCR Primer PE 2.0 from Illumina with Phusion DNA Polymerase were involved in the amplification approach to enhance the purified cDNA template. Finally, using the Illumina GAIIx platform, cDNA library products were sequenced on a paired-end flow cell after validation was completed on a Bioanalyzer.

### *De novo* Assembly

Preprocessing and filtering of the original reads obtained from sequencing were made prior to the *de novo* transcriptome assembly by the Illumina platform in order to avoid sequencing errors. The first step was the removal of raw reads through filtration by Illumina’s Failed Chastity software that eliminates all reads with a chastity score of >0.6 on the first 25 cycles. Chastity score is a quality control measure, where the read quality score is linked to the intensity of a base signal, designed to remove poor quality reads, which have several ambiguous base calls. Next, the removal of raw reads with adaptor corruption and indistinct trace peaks or “N” in the sequence trace was made. Finally, raw reads with more than 10% of Phred-scaled probability (Q) bases which are less than 20 were eliminated.

OASES software^19^ was used to generate contigs from the resulting short reads that successfully overlap with others, using a Kmer size of 47 to generate contigs with N50 of 661 bp. In a two-step process, we performed the assembly of contigs generated by Velvet for the second trimmed data set (k1/4 47) into transcripts using Oases with default parameters. By mapping the clean reads back to the corresponding contigs based on their paired-end information, the identity and distance can be recognized. Scaffolds were generated once the contigs and the gap between them were filled using “Ns”. Lastly, the most complete scaffolds were then filled with paired-end clean reads based on the complementary sequences to the scaffolds. This resulted in sequences with a minimum number of Ns that also could not be more prolonged at either end.

### Cell Disruption Using AFA Processing

MD2 pineapple leaves were ground in 1–10 mg of liquid nitrogen and transferred into a Covaris tube (part number 520096-microtube AFA Fiber Screw-Cap 6 × 16 mm) and 450 μL of distilled water with 50 μL of TCA and 5 μL of 0.5 M dithiothreitol (DTT) was added into the tube.

Next, the tube was processed using an ultrasonicator S220 from Covaris, through a process known as AFA (adaptive focused acoustics), to begin plant cell disruption. The settings were as follows: 20% duty cycle, intensity = 8, cycle per burst = 450, time = 420 s. Subsequently, the sample was transferred to a new 2 mL tube and was frozen in −20 °C for 45 min. Next, the tube was spun for 20 min at 30,000*g* before the supernatant was discarded and the green pellets were retained. 2000 μL of ice-cold acetone with 5 mM DTT was added and the pellets were suspended gently using a pipet to aspirate. The suspension was kept frozen at −20 °C for another 45 min followed by centrifugation at 30,000*g* for 20 min and then the supernatant was discarded. These steps were repeated, and finally, the pellet was left to dry for ten min at room temperature.

The pellet obtained was weighed using a fine balance before 50 μL of 4% SDS with 5 mM DTT was added for every 1 mg of sample. IAM was added to a final concentration of 15 mM before the mixture was incubated for 20 min in order to alkylate the cysteines. The reaction was stopped by adding 5 mM DTT. The tube was spun down again at 3000*g* for 5 min to obtain the protein. Protein concentration was determined using a direct detect spectrometer at AM3 using 4% SDS with 5 mM DTT as a blank.

### Filter-Aided Sample Preparation (FASP)

25 μg of MD2 protein and 200 μL of UA2 (urea extraction buffer 2) were transferred into a new spin tube and spun down at 14,000*g* for 15 min at 20 °C. The step was repeated again with 100 μL. To alkylate the samples, 50 μL of UA1 buffer with 0.05 M iodoacetamide was added to the filters, and the samples were incubated in darkness at room temperature for 30 min.

Centrifugation was done again with the IAM solution, and the filters were washed twice with 100 μL of UA2 buffer followed by a further two washes of UA3 buffer. 50 μL of UA3 buffer was added to the filter, and the protein was digested first using endoproteinase LysC at an enzyme/protein ratio of 1:50 at 37 °C for 3 h and a fresh collection tube was used for subsequent spins (10 μL of a 100 ng/μL LysC solution was added; there was 50,000 ng of protein present). Following this, the solution was diluted to 200 μL with the addition of 150 μL of 50 mM Tris-HCl (pH 8.5) and the protein was further digested with trypsin at a protein/enzyme ratio of 1:100 overnight at 37 °C (10 μL of a 50 ng/μL trypsin solution was added).

After digestion, peptides were collected by centrifugation at 4000*g* at 20 °C for 15 min and the filtration units were washed once with 50 μL of UA1 buffer and subsequently with two 50 μL washes of 40 mM ammonium bicarbonate. Peptides were cleaned up with R3 beads, lyophilized, and stored dry at −20 °C until analysis.

### Peptide Desalting (96-Well Format)

One mg (100 μL of 10 mg/mL stock) of POROS R3 beads was added to each well (labeled each one per sample) in a Corning 96-well plate. The plate was centrifuged at 200*g* for 1 min and this step was repeated once again. Next, 50 μL of wet solution was added, followed by gentle resuspension before the centrifugation was repeated. Again, this step was repeated with substitution of 50 μL of wash solution and the flow through was discarded. The filters were removed from FASP tubes and 100 μL of the protein sample was added to the corresponding well followed by a gentle resuspension and was centrifuged again at 200*g* for 1 min and 100 μL of the sample was added. This step was repeated until all samples were added and washed with wash solution followed by centrifugation twice.

50 μL of elution solution was added and was spun again at 200*g* for one min and repeated. The eluted sample was transferred into chromatography sample vials and was dried in a Heto SpeedVac for 2 h. Ten μL of 5% acetonitrile with 0.1% formic acid was added to suspend the dried peptides. The samples were ready to be used for LC/MS, with the necessity of dilution with 5% acetonitrile and 0.1% formic acid solutions.

### Mass Spectrometry

All MD2 pineapple protein extractions and mass spectrometry were performed in the Bio-MS Research facility, Faculty of Biology, Medicine, and Health, University of Manchester. Digested samples were analyzed by liquid chromatography-mass spectrometry (LC–MS)/MS using an UltiMate 3000 Rapid Separation LC system (RSLC, Dionex Corporation, Sunnyvale, CA) coupled to an Orbitrap Elite (Thermo Fisher Scientific, Waltham, MA) mass spectrometer. Peptide mixtures were separated using a gradient from 92% A (0.1% FA in water) and 8% B (0.1% FA in acetonitrile) to 33% B, in 104 min at 300 nL min^–1^, using a 75 mm × 250 μm i.d. 1.7 M CSH C18, analytical column (Waters). Peptides were selected for fragmentation automatically by data-dependent analysis.

### Quality Control of MS Spectra

An XIC (extracted ion chromatogram) filtering process was performed on the selected peptides. Several aspects were given attention to remove the noise, such as the peak sharpness, signal significance level, signal-to-noise (S/N) ratio, triangle signal area similarity, and local signal corresponding to the local zigzag index.^20^ The filtered raw spectra were then converted to a commonly accepted format for database searching, mgf (Mascot Generic Format), using MsConvert,^21^ prior to the search against the database.

### V3 and MD2 Pineapple Genome Database

**Figure 1:**
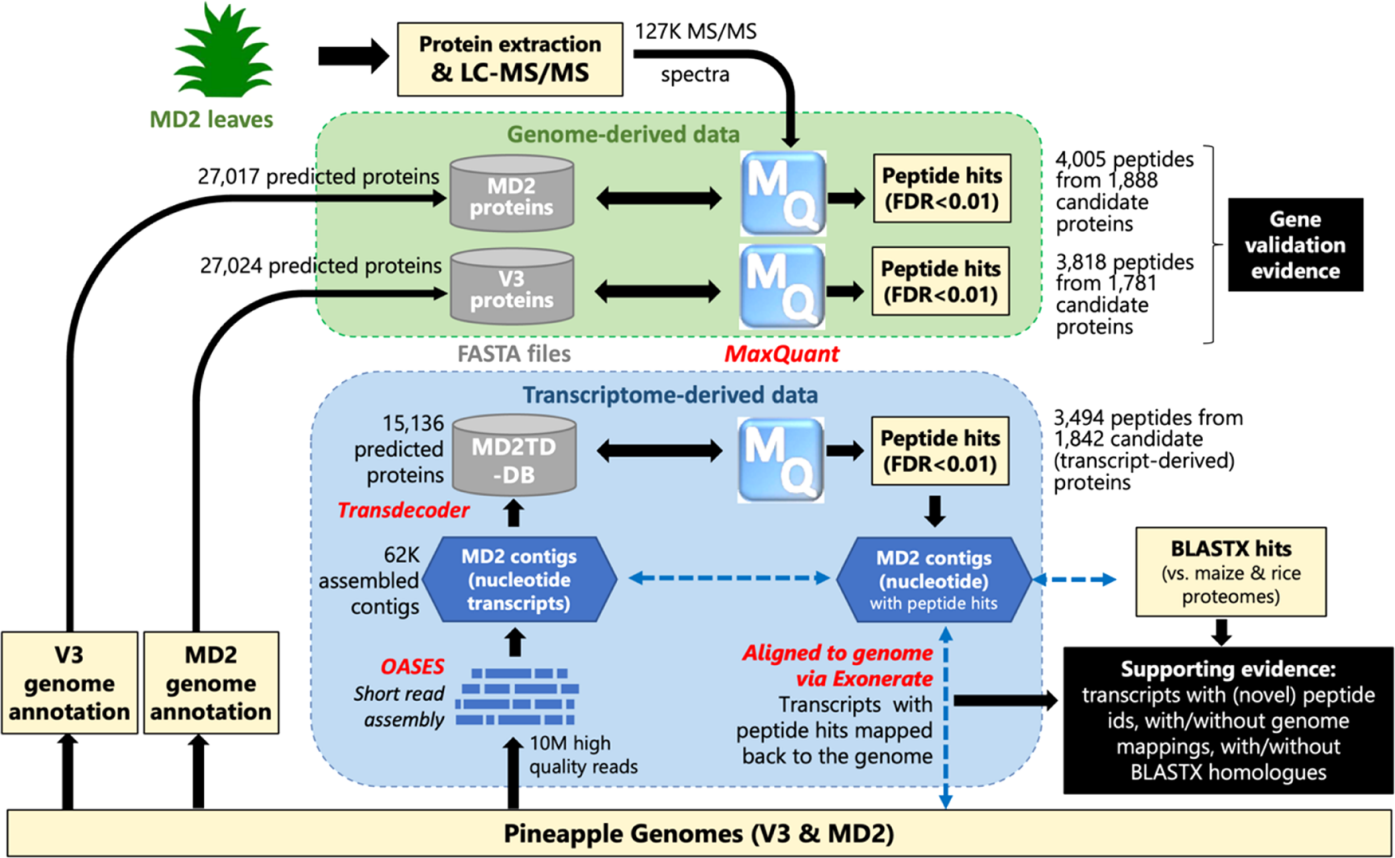
Workflow of the proteogenomics pipeline. The flow diagram shows how transcriptome-predicted proteins are integrated in the proteogenomic pipeline and compared to protein searches derived from genome annotations to identify a candidate novel gene structure. Supporting evidence from contigs that contain novel MS-detected peptides suggests a novel gene structure: if the contig matches with the pineapple genome, it could align to an existing gene and hence be explained by novel gene structure or variants (since the peptide sequence is novel) or could align to the genome in a gene-free region (and be a novel gene annotation). Equally, unaligned contigs with peptide evidence may represent genes not currently in the sequenced genome; in these cases, BLAST evidence of homologues in other plant genomes offers supporting evidence that they are protein coding.

To benchmark peptide identifications from the MD2 pineapple transcript-derived contigs, existing genome annotations were obtained. First, the V3 set annotated 24,063 complete and a further 2,961 partial genes, covering a total of 27,024 predicted protein-coding genes. Second, the MD2 pineapple draft genome appeared in 2016.^16^ Relevant genome and annotation statistics are listed in Figure 1.

### Database Preparation for the Target-Decoy Approach Using MD2 Pineapple Translated Contigs

MD2 pineapple transcript-derived contigs were translated into protein sequences using the Transdecoder v.5.0.0^3^ program. Transdecoder is written to detect protein-coding regions based on the composition of nucleotides/codons with a minimum length of open-reading frames in the transcript sequences and was run using default parameters. From the 62,002 assembled contigs, Transdecoder predicted 15,366 putative protein sequences. These putative protein sequences were used as the database against which the acquired MD2 pineapple spectra were searched.

### Database Search for Peptide Identifications Using MaxQuant

MD2 spectra were searched against three different databases: a database derived from the translated transcript and the two reference pineapple genome annotations, V3 and MD2, used as a benchmark for the searches.^15,16^ MaxQuant^22^ (version 1.6.3.4) was run independently for each different database using MaxQuant’s reversed decoy data set and inbuilt set of known contaminants. Default search parameters were used with standard tryptic digestion, allowing two missed cleavages and minimum peptide lengths of six. Carbamidomethyl cysteine was specified as a fixed modification. Oxidation of methionine, N-terminal protein acetylation, and phosphorylation of serine, threonine, and tyrosine were specified as variable modifications. MaxQuant’s “match between runs” option was enabled, and searches were constrained to 1% false discovery rate (FDR) at all levels. Additional searches using ProteomeXchange data set PXD010375, collected from *A. comosusvar. bracteatus*, were performed against the same three databases using identical search parameters to those supplied by the authors.^18^

### Contigs and Peptide Remap to the V3 Pineapple Genome

The contigs derived from the assembly of the MD2 pineapple transcriptome were aligned back to the V3 pineapple genome in order to locate the coordinates of the coding sequences. The EXONERATE v2.2.0^23^ program was used for this purpose. The parameters for the program were as follows: “—model est2genome, −score 2000, and −percent 95”.

### Detection of Novel Protein-Coding Regions

Identification of putative novel genes or novel gene structures for the MD2 pineapple was done by comparison of GTF coordinates of the peptides and MD2 contigs with the pineapple genes encoded in the genome. Novel peptides or their parent contigs which overlapped or are entirely contained within the range of an annotated gene on the V3 genome sequence were categorized as “refined gene structures”, since the most likely explanation is that they differ from the existing V3 annotation set, but nevertheless map to existing genes. When no overlap with annotated genes was found, these cases were investigated further and were categorized as “putative novel genes” for the V3 pineapple genome.

### Validation of Novel Genes

The NCBI BLASTX and BLASTP tools were used to validate the discovery of novel genes using the proteogenomics pipelines used in this study. MD2 pineapple contigs were aligned against the annotated pineapple V3 protein database using BLASTX tools (https://blast.ncbi.nlm.nih.gov). For the identified peptides from the database search, alignment against the pineapple protein database was carried out using the BLASTP tool. The parameters for retaining the significant matches were as follows: *e*-value < 1 × 10^–12^, % identity >70%, and aligned coverage >90%. For each relevant contig, a BLASTP search was performed against the proteomes of the V3, the MD2, and the more recent F153 pineapple (genome assembly ASM154086v1), as well as the proteomes of rice (IRGSP-1.0), maize (Zm-B73-REFERENCE-NAM-5.0), and *Arabidopsis* (TAIR10), downloaded from Ensembl.

MSA (multiple sequence alignment) analysis^24^ was done using ClustalW^25,26^ to visualize the homology as evidence of the novelty of the genes discovered in the MD2 pineapple. The TDMD2 contigs and associated peptides identified as being novel relative to V3 and MD2 were mapped to the ASM154086v1 version of the F153 pineapple genome. The contig mapping was achieved using EXONERATE in the same manner specified above to map the cation to the V3 genome. The coordinates and associated information were extracted and converted into bed file format in order to allow them to be hosted as a custom Gene Track on the Ensembl Plants to visualize the genes affected.

## Results and Discussion

### Sequencing and Assembly of MD2 Pineapple Transcriptome

The purpose of this study was to evaluate a proteogenomics approach to improve the pineapple genome annotation using an MD2-assembled transcriptome as a search database. Following RNA extraction from MD2 pineapple leaves, two sets of cDNA libraries were sequenced, generating 23,717,364 paired-end raw reads with lengths of 75 bp, reduced to 10,071,725 high-quality reads with 99.30% Q20 bases (base quality more than 20) after quality control to also remove rRNA. Subsequent assembly using Oases^19^ resulted in a total number of 62,002 transcript sequences. This assembly was generated prior to the availability of either genome, demonstrating that short-read *de novo* transcriptome assembly can be achieved for a newly sequenced crop plant, MD2 pineapple, despite the lack of scaffolds in public databases.^27^ We retained this “pregenome” assembly as our basis for proteogenomic experiments to avoid bias and to demonstrate its utility in proteogenomics. The 62,002 transcripts were then translated into protein using Transdecoder,^3^ generating 15,366 predicted proteins, which were used as a search database for protein identifications.

### Identification of Peptides from the Annotated V3 and MD2 Pineapple Genome

Only the V3 genome was available at the outset of this study, and initial comparisons of transcriptome-derived proteomics were made only with the relatively immature V3 annotation released in 2015. Although this genome annotation benefitted from *de novo* transcriptome sequencing, many polyadenylated reads are often found unaligned and such annotations can have potential limitations.^28,31^ A proteogenomics approach can add value for newly annotated genomes with experimental evidence at both the transcriptional (cDNA) and translational (protein) level.

In this study, six biological replicates generated total proteins extracted from leaves were subject to LC–MS/MS and generated 127,154 MS/MS spectra. MaxQuant searches against the protein database derived from the V3 pineapple genome were filtered at a 1% FDR threshold yielding 3,818 peptides, which in turn support 1781 attendant proteins. Subsequently, in comparison, 4,005 peptide identification supported 1,888 proteins from searches against the more recent MD2 genome database (Table 1). Collectively, these identifications represent validation evidence for the genes predicted from the corresponding genomes, as indicated in Figure 1.

**Table 1:** Total Number of Peptides and Proteins Identified by MaxQuant at 1% FDR

database	total unique peptide identifications	total predicted proteins
V3 pineapple genome (Ming et al.^15^)	3818	1781
MD2 pineapple genome (Redwan et al.^16^)	4005	1888
MD2TD-DB translated contigs	3494	1842

### Peptide Identification from MD2 Transcript-Derived Contigs (MD2TD Transcripts)

**Figure 2:**
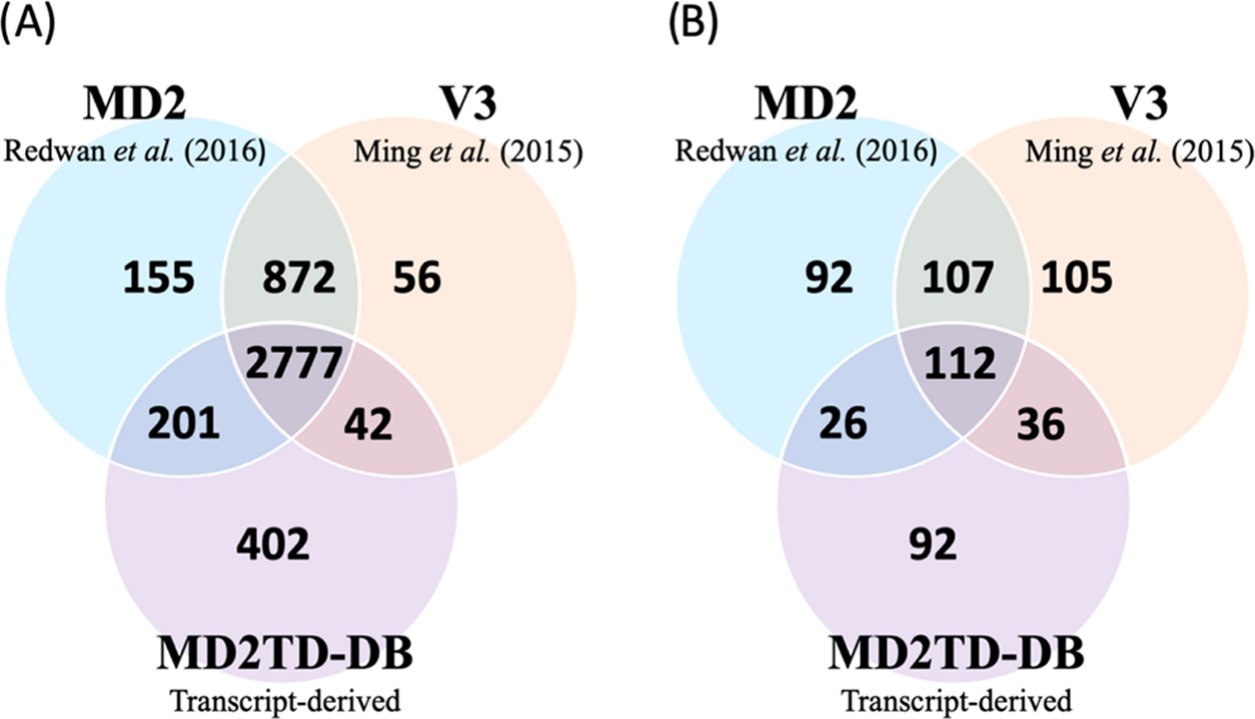
Venn diagrams of peptide identifications. (A) Identified peptides between database searches against the V3 genome (Ming et al.^15^), MD2 genome (Redwan et al.^16^), and the translated MD2 transcript-derived contigs. (B) Identified peptides from the TMT data set of *A. comosus* var. bracteatus (ProteomeXchange ID PXD010375), between database searches against the V3 and MD2 genomes, as well as the translated MD2 transcript-derived contigs. All peptide and parent protein searches were filtered at a 1% FDR.

Searches against the putative protein sequences predicted from the assembled MD2 contigs yielded a total of 3,494 peptides and 1,842 candidate proteins at the same FDR. We compared the identified peptides against the two sets from the pineapple genomes: V3^15^ and MD2 pineapple^16^ (Figure 2). As expected, a high fraction of peptides are shared, totaling 2777 across all three databases, with the largest single value identified in the most recent MD2 genome.^16^ This reassuring level of overlap suggests that high-quality peptide identifications have been derived from all three annotation sources, which map to 1460 unique V3 genes.

Although most peptides/proteins are derived from the two genome annotations, significant numbers of peptides (and putative parent proteins and hence genes) were identified exclusively from the transcript data (Figure 2). First, 201 peptides were identified in the MD2 transcript-derived (MD2TD) database, missed in the original V3 genome annotation, but subsequently identified from the latter MD2 genome annotation.^16^ Additionally, 402 peptides were unique to the MD2 transcript-derived (MD2TD) database. These novel peptides could represent novel genes/gene structures, paralogues, novel splice variants, or polymorphisms, therefore requiring further investigation, as indicated in the bottom right of Figure 1. All data relating to these peptides are provided in Table S1 for use by the community.

### Mapping the MD2TD-Derived Contigs against the V3 Pineapple Genome Revealed Misannotation Events in the Genome Annotations

**Figure 3:**
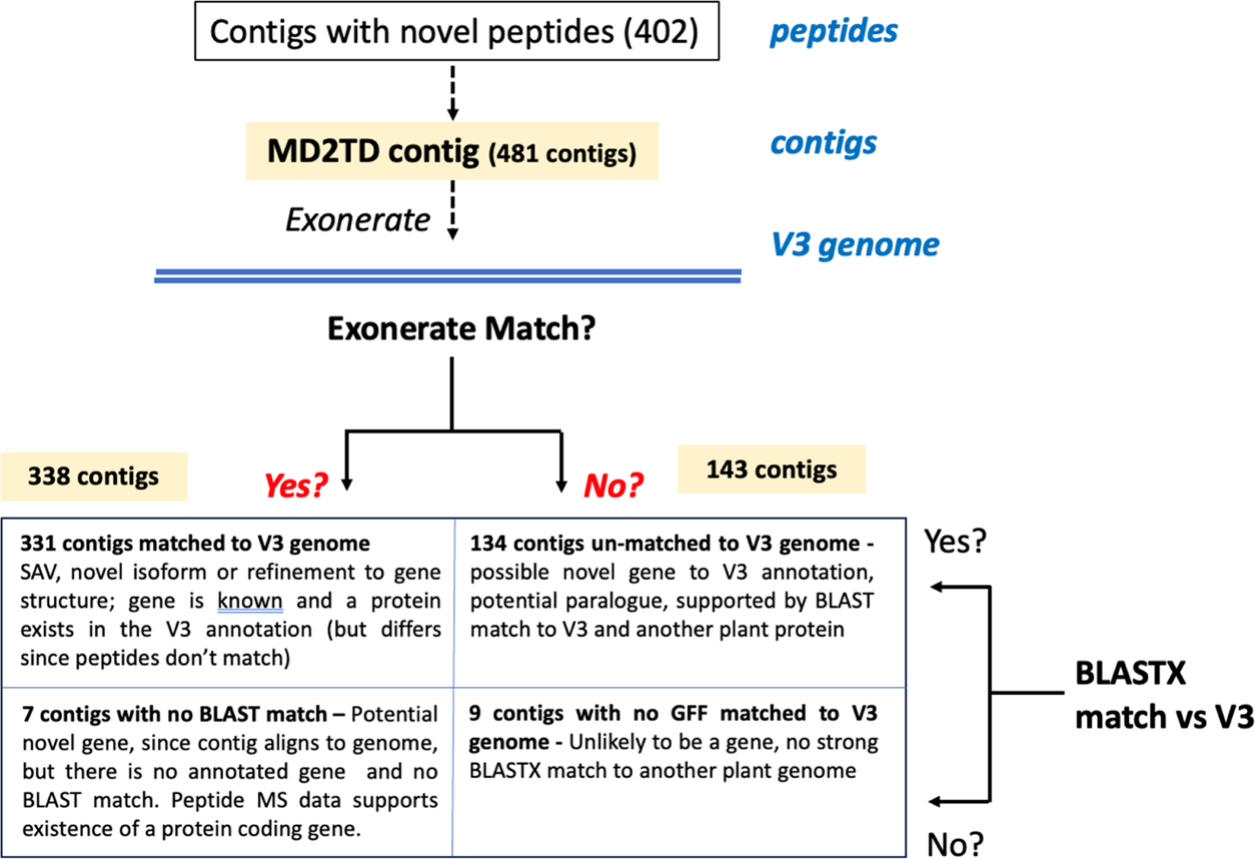
Flow diagram of peptide-supported contig annotations. MD2 contigs with novel peptide hits exclusive to this transcript-derived set were compared to the V3 genome using Exonerate and the V3-predicted protein sequences using BLASTX.

The MD2TD contig nucleotide sequences were searched against the V3 pineapple genome sequence, using the open source, splicing-aware, mapping tool EXONERATE v2.2.0,^23^ using the “est2genome” model. It has been proven to be a reliable tool in aligning ESTs and short reads as low as 20 bp to rice genomes and producing high-quality gene data sets.^32^ The tool does not have a statistical scoring framework, so alignments were selected on the basis of having scores above 2000, at least 95% identity, and 50% query sequence coverage. Using these search criteria, 481 MD2TD contigs associated with the 402 unique peptides were searched against the V3 genome, yielding 360 high-quality alignments from 338 contigs (Figure 3). 331 of these contigs overlapped or were within annotated genes from the Phytozome set, suggesting potential revisions to the annotated gene structure since they contained novel peptide sequences. The other 7 contigs did not map to genic regions, but all had good BLASTX hits to V3 genes and much stronger BLAST matches with MD2 genes, consistent with the improved annotation.

All 481 contigs were also searched using BLASTX against the V3, MD2, and F153 pineapple proteomes, as well as rice, maize, and *Arabidopsis* proteomes to establish their similarity, with all bar 12 showing sequence similarities with either another pineapple, *Arabidopsis*, rice, and/or maize gene with good BLAST scores (*E*-values < 1 × 10^–30^). All data are provided in Table S1 and a number of example MSAs are provided in the Supporting Information (Figures S1–S5). For example, Contig Locus 1660 matches orthologues of the DEAD-box, ATP-dependent RNA helicase 53 (Figure S3). The V3 sequence has a lower shared identity than any of the other sequences, even the distant relative *Arabidopsis*,^33^ but shares inferred GO terms. Additionally, the V3 BLAST match (Aco011096.1) is found on chromosome LG04, whereas the Exonerate alignments both map to chromosome LG09. This LG04 gene could therefore represent a paralog.

143 MD2-dervied contigs did not align to the V3 genome annotation, but 134 of these contigs have significant BLASTX matches with some or all of the other plant proteomes. For example, in Figure S5, Locus 2410 transcript 4 does not align to V3 or match a F153 protein but does match with others, including an ortholog of the cytochrome c oxidase subunit 2, COX2, in *Arabidopsis*. COX2 is known to exist and is vital for development in plants, but it possesses a multicopy complex evolutionary history with both nuclear and mitochondrial lineages, existing at the same time in many plant species.^34−36^ Other locus 2410 transcripts successfully align to the V3 genome overlapping the same gene, but these also still align more closely to the *Arabidopsis* mitochondrial sequence than to the nuclear pineapple genome sequences. Further evidence would be needed to unambiguously resolve this, but this highlights how the protein support for transcripts can be used to inform genome annotations.

In addition to a novel gene structure, a proteogenomics pipeline can be used to discover potential single amino acid variants (SAVs), and given the high heterozygosity within the pineapple genome,^15^ we expected to uncover some. Of 280 contigs with MD2 BLAST matches of 100–99% percent identity, 106 sequences possessed a single mismatch or an alignment 1 base short of the total length of the contig, permitting two mismatches to add an extra 43 contigs, for a total of 53% of contigs assessed being potential SAV candidates. In order to provide additional external validation of the novel contigs, we used MaxQuant to search a publicly available, ornamental pineapple data set against the genomes used in this analysis (Figure 2b). Despite being a distant, noncrop relative, we still discover peptides that match each genome annotation, both shared and unique; we identify 36 that independently support the novel contigs, as summarized in Table S1.

### Mapping the MD2TD-Derived Contigs against the F153 Pineapple Genome Shows Variation in Aco010232.1—A Key Element Required for CAM

The V3 pineapple genome is still at a relatively early stage of development. Since its debut in 2015,^15^ the genome annotation, sequence, and RNA-Seq data have been publicly released via the Comparative Genomics Web site (CoGe)^29^ in Phytozome^30^ but it has been superseded more recently with an F153 genome in Ensembl Plants,^31^ as well as the MD2 pineapple draft genome that was published in 2016.^16^ Given this progression in the pineapple genome annotation, we evaluated our proteogenomics pipeline by mapping MD2TD contigs with MS-based peptide evidence to the more recent F153 annotation to examine potential genomic variation between our MD2-based data and the F153 sequence. Of the 481 novel contigs, we find 281 contigs with below 100% percentage identity or missing a BLAST match in the current F153 genome annotation (Supporting Information, Table S1).

**Figure 4:**
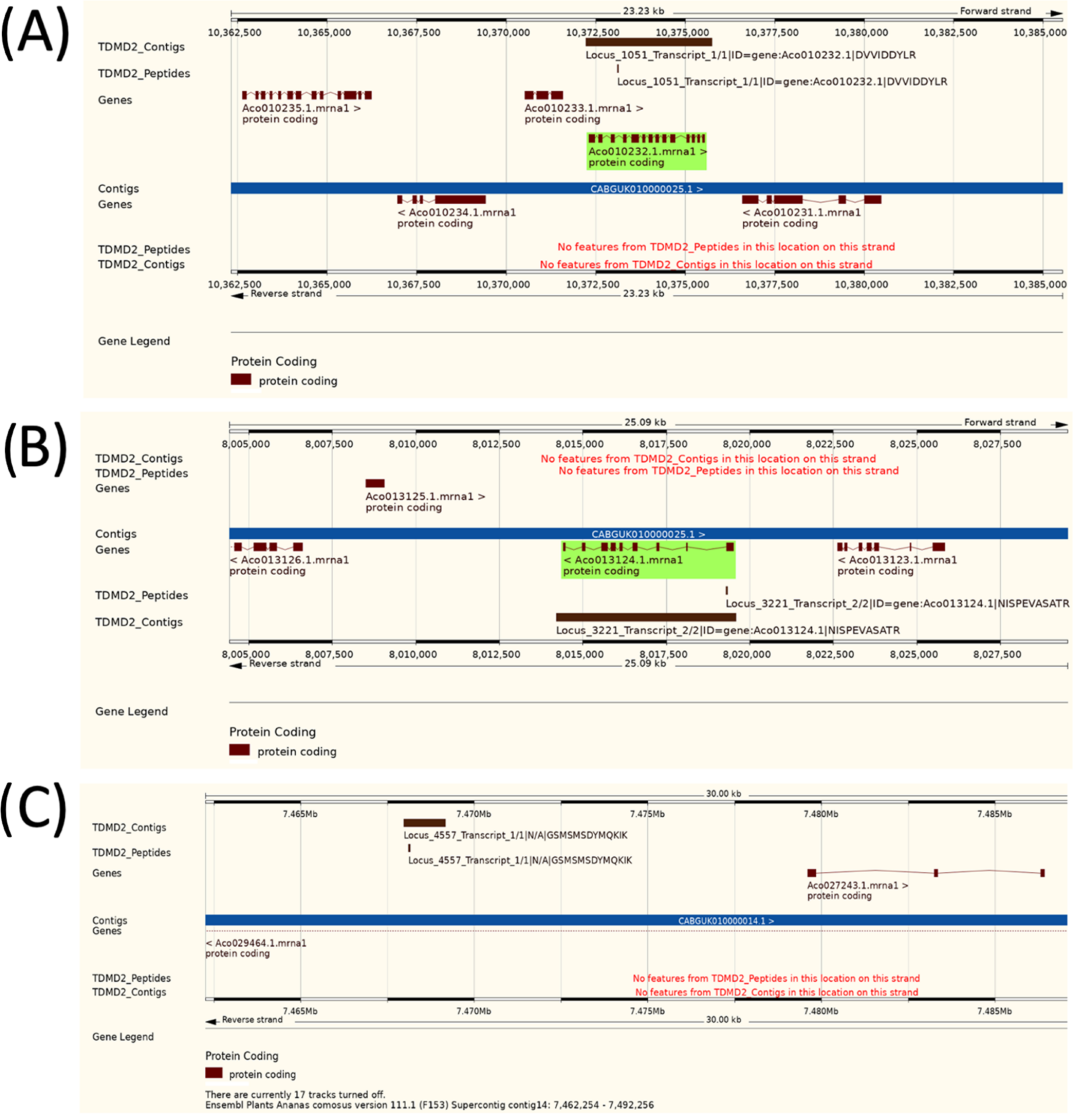
Custom Gene Tracks, for example, TDMD2 contigs and peptides aligned to the F153 genome. Gene Track view of the alignment of (A) Locus 1051, (B) Locus 3221, and (C) Locus 4557 to the F153 genome exported from the Ensembl Plants Region Viewer. Locus 1051 aligns to the forward strand of contig 25 from the F153 pineapple genome and matches with the gene Aco010232.1. Locus 3221 aligns to the reverse strand of contig 14 from the F153 pineapple genome and matches with the gene Aco013124.1. Locus 4557 aligns to the forward strand of contig 17 from the F153 pineapple genome and matches with no genes.

In order to make this data available to all, we aligned the contigs with EXONERATE to the F153 genome and mapped the coordinates of the alignments and the position of the novel peptides within the alignments, generating a custom Gene Track for viewing on the Ensembl Plants viewer (see Supporting Information, Files S2 and S3). Figure 4 shows Locus 1051 aligning to the F153 genome overlapping with Aco010232.1, which is a copy of malate dehydrogenase (MDH). The alignment looks robust with sensible genetic architecture but is not a perfect match; the source of this variation may be strain-specific differences between F153 and MD2. It is interesting that the variation could be identified in MDH as it is central to metabolism of eukaryotes generally, and especially important in the proper functioning of leaf cells in plants with Crassulacean acid metabolism, but it is known that there are multiple copies of MDH in pineapple, perhaps explaining the lack of constraint.^15^ How Locus 4557 aligns to the F153 genome is also shown in Figure 4 as an example of a contig that aligns but does not match a region with a gene. Locus 4557 is an unusual case that aligns in both V3 and F153 at multiple locations in each genome. Based on the BLAST matches from the V3 alignment, it appears to be a transposable element; consistent with the small size of the contig and presence at multiple genomic locations, thousands of retrotransposons are known to be present in the pineapple genome.^15^

## Conclusions and Perspective

The limitations of *ab initio* gene prediction methods have long been discussed in the literature.^37−40^ Here, we used translated transcripts of the MD2 pineapple in a proteogenomics pipeline to support and improve the newly introduced crop pineapple genome annotations, comparing it against the original V3 genome,^15^ the updated MD2 genome,^16^ and the more recent F153 genome.^31^ In addition, to deriving direct proteomic evidence supporting ∼1800 pineapple genes, we identified 402 peptides that were unique to a set of MD2 transcript-derived proteins (Figure 2). These peptides match with 481 contigs, of which 331 also align to the V3 pineapple genome and 330 align to the F153 pineapple genome. These partial matches with known genes make this category strong candidates for novel gene structure, where the gene annotations need to be expanded to encompass the rest of the contig. A further 7 contigs containing novel proteogenomic-supported peptides were not aligned to V3, and 6 of them possess strong BLAST hits to known plant proteins, including excellent matches (identical for three of them) to proteins in the improved reannotation of the MD2 genome.^16^ This demonstrates how prompt use of transcriptomics can validate and improve gene annotations, forming part of a mounting body of evidence for use of proteogenomics in improving genome annotations.^12−14,41^ The complementary use of proteogenomics pipelines can improve sensitivity and precision compared to the direct *ab initio* translation methods alone by allowing the identification of more PSMs, peptides, and proteins.

## Data Availability

Mass spectrometry data have been deposited with PRIDE, under the identifier PXD045998, including all raw files, Maxquant output, and Fasta files searched.

## Supporting Information Available

MSA of a potential novel transposable element, with novel MS-supported peptides (Figure S1); multiple sequence alignment (MSA) of Locus_7918_Transcript contig translation with plant protein homologues (Figure S2); multiple sequence alignment (MSA) of a potential novel paralog supported by novel peptides (Figure S3); MSA of a potential novel paralog supported by novel peptides (Figure S4); MSA of Locus_7918_Transcript contig translation with plant protein homologues (Figure S5) (PDF)
Contigs matches to BLAST hits of V3, F153, and MD2 pineapple genomes, as well as Arabidopsis, rice and maize genomes, also incorporating peptide matches found in MaxQuant searches of publicly available *Ananas comosus* var. bracteatus data (PXD010375) (XLSX)
Bed file of Contigs mapped to the F153 Pineapple Genome hosted on Ensembl Plants region, can be used to create a Custom Gene Track of the results of this study (TXT)
Bed file of Peptides mapped to the F153 Pineapple Genome hosted on Ensembl Plants region, can be used to create a Custom Gene Track of the results of this study (TXT)
