# Supplementary material for: Proteogenomic Gene Structure Validation in the Pineapple Genome
Source: J Proteome Res. 2024 Apr 23;23(5):1583–92. doi: 10.1021/acs.jproteome.3c00675 (PMC11077482; doi:10.1021/acs.jproteome.3c00675)
Supplement: Supplementary file 1 — pr3c00675_si_001.pdf [file pr3c00675_si_001.pdf]

Supplementary Material

Proteogenomic gene structure validation in the pineapple genome

Norazrin Ariffin[1,2*,†], David Wells Newman[1,†], Michael G. Nelson[1], Ronan O'cualain[1], Simon J Hubbard[1*]

1. School of Biological Sciences, Faculty of Biology Medicine and Health, MAHSC, University of Manchester, Michael Smith Building, Oxford Road, Manchester M13 9PT, United Kingdom.
2. Department of Agriculture Technology, Faculty of Agriculture, Universiti Putra Malaysia, 43400 Serdang, Selangor Darul Ehsan, Malaysia.

*Corresponding Authors:

Simon J Hubbard: School of Biological Sciences, Faculty of Biology Medicine and Health, MAHSC, University of Manchester, Michael Smith Building, Oxford Road, Manchester M13 9PT, United Kingdom.

Norazrin Ariffin; Department of Agriculture Technology, Faculty of Agriculture, Universiti Putra Malaysia, 43400 Serdang, Selangor Darul Ehsan, Malaysia;

[†] Should be considered equal first authors

Author contact details:

Norazrin Ariffin	email: norazrin@upm.edu.my
Simon J Hubbard	email: simon.hubbard@manchester.ac.uk

Table of Contents

Figure S1. MSA of a potential novel transposable element, with novel MS-supported peptide. S2

Figure S2. Multiple Sequence Alignment (MSA) of Locus_7918_Transcript contig translation with plant protein homologues. S3

Figure S3. Multiple Sequence Alignment (MSA) of a potential novel paralog supported by novel peptides S4

Figure S4. MSA of a potential novel paralog supported by novel peptides. S4

Figure S5. MSA of Locus_7918_Transcript contig translation with plant protein homologues S5

Table S1: Contigs matches to BLAST hits of V3, F153, and MD2 pineapple genomes, as well as Arabidopsis, rice and maize genomes, also incorporating peptide matches found in MaxQuant searches of publicly available *Ananas comosus var. bracteatus data (*PXD010375*),* (XLSX)

File S1 - TDMD2ContigsCurrentVersion.bed: Bed file of Contigs mapped to the F153 Pineapple Genome hosted on Ensembl Plants region, can be used to create a Custom Gene Track of the results of this study. (TXT)

File S2 - TDMD2PeptidesCurrentVersion.bed: Bed file of Peptides mapped to the F153 Pineapple Genome hosted on Ensembl Plants region, can be used to create a Custom Gene Track of the results of this study. (TXT)

Figure S1. MSA of a potential novel transposable element, with novel MS-supported peptide.

Transcript “Locus_4557_Transcript” aligns to the V3 pineapple genome at an unannotated locus and returns a BLASTX match with a single amino acid variant (SAV) that maps to a different chromosome than the alignment. It has a stronger BLAST match to the F153 annotation than the V3 genome and matches to transposable elements in the other plant species. Aligned MS-supported peptide regions are highlighted in yellow, with amino acid substitutions in the homologues shown in turquoise and missing sequence in red. E-values for each of the candidate proteins are shown at the left side.

Locus 4557. possesses a single transcript that matches to an uncharacterized protein in the pineapple genomes, and also has weak BLASTX matches to an integrase catalytic core domain containing protein in rice and a Copia-like polyprotein/retrotransposon in Arabidopsis. The Copia-like polyproteins contain integrase domains themselves so it seems possible that these sequences may be related retrotransposons, albeit distantly in some of the cases [34]. This locus shares more similarities to the MD2 BLASTX match than the V3 genome, suggesting that the V3 BLASTX match originates from elsewhere in the genome. Indeed, the gene ID Ac022997.1 maps to chromosome LG06 while Locus 4557 aligns to chromosome LG03.

	E-value	Sequence
	1.30×10^{-8}	Os02t0671800-01
	3.67×10^{-23}	AT1G34070.1
V3 annotation	2.4×10^{-149}	Aco022887.1
		Gene.7852::Locus_4557_Transcript_1/1
F153	2.4×10^{-155}	XP_020101576.1

```
Os02t0671800-01                        VMNTRMALTATKKGNLKVAEYVAKMRGLADDMASAGKKLDDDDIVSDILAGLDDDFDPVV
AT1G34070.1                            ALRLDSELRTKDIGDMRVADYYRKMKKLADSLRNVDVPVTDRNLVMYVLNGLNPKFDNII
Aco022887.1                            AFSLKSELWSIKKGSMSMSDYIQKIKTIGNALQAIGEIESDHNLVMTVLLGLPEEYRGFV
Gene.7852::Locus_4557_Transcript_1/1   AFSLKSELRSIKKGSMSMSDYMQKIKTIGNALQAIGEIESNHNLVMTVLLGLPEEYRGFV
XP_020101576.1                         AFSLKSELRSIKKGSMSMSDYMQKIKTIGNALQAIGEIESDHNLVMTVLLGLPEEYRGFV
                                       .:     * : . *.: :::*  *:: :.: :   .    : ::*  :* **  .:  .:

Os02t0671800-01                        SAVAQRSEPISVGELFSQLVTFEQRLELRQGGIQ---------------------SSTNS
AT1G34070.1                            NVIKHRQPFPSFDDAATMLQEEEDRLKRAIKPNPTHVDHSSSSTVLACSEAPPVT---NF
Aco022887.1                            SALNTHRNKPTCEQFRPLLMQEETEVQRRTSVTT-------------SSAIPTIDGEALY
Gene.7852::Locus_4557_Transcript_1/1   SALNTHRNKPTFEQLRPLLMQEETEVQRRTSVTT-------------SSAI---------
XP_020101576.1                         SALNTHRNKPTFEQLRPLLMQEETEVQRRTSVTT-------------SSAIPTIDSEALY
                                       ..:  :    :  :    *   * .::
```

Figure S2. Multiple Sequence Alignment (MSA) of Locus_7918_Transcript contig translation with plant protein homologues.

Figure S2 shows a further example, with an MD2 transcript-derived protein sequence from Locus 7918 which contains the novel peptide “ASTGIYDDLKPR”. The contig aligned to an unannotated region of the V3 genome on the “LG10” chromosome. The BLASTX results revealed the contig was homologous to orthologues of putative acetyl-CoA acetyltransferase, cytosolic 2 (ACAA2), an important enzyme in the metabolism of fatty acids. Plants that have crassulacean acid metabolism (CAM) depend heavily on the activity of ACAA2 in preserving water in arid conditions. This is related to carbon dioxide stored in malate form and during the day, malate is decarboxylated to release the CO2 for photosynthesis process.

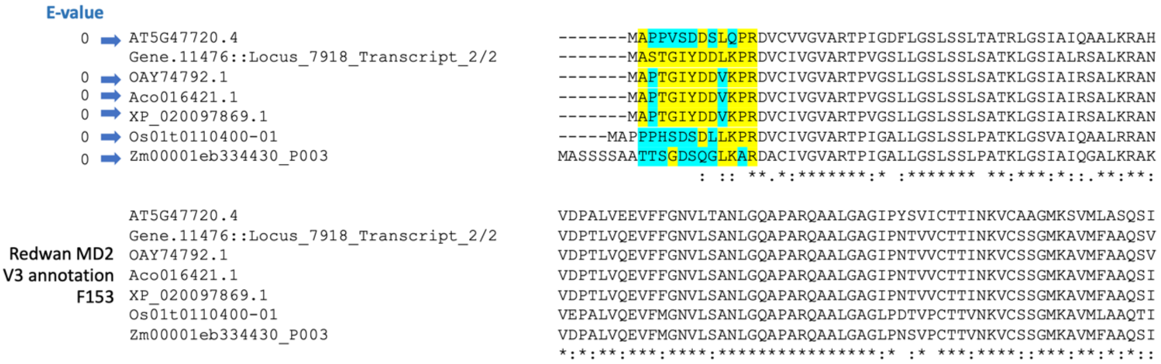

Figure S2. Multiple Sequence Alignment (MSA) of Locus_7918_Transcript contig translation with plant protein homologues. The transcript “Locus_7918_Transcript” aligns to the V3 genome at an unannotated locus, has two MS-detected peptides, and possesses homologues in 3 plant proteomes. The aligned MS-supported peptide region is highlighted in yellow, with amino acid substitutions in the homologues shown in turquoise and missing sequence in red. BLASTX E-values of the transcript versus each of the candidate proteins are shown left.

Two further example alignments showing contig-predicted proteins and their MS-supported peptides aligned with homologous protein sequences from pineapple genome annotations and other plant genomes.

Figure S3. Multiple Sequence Alignment (MSA) of a potential novel paralog supported by novel peptides

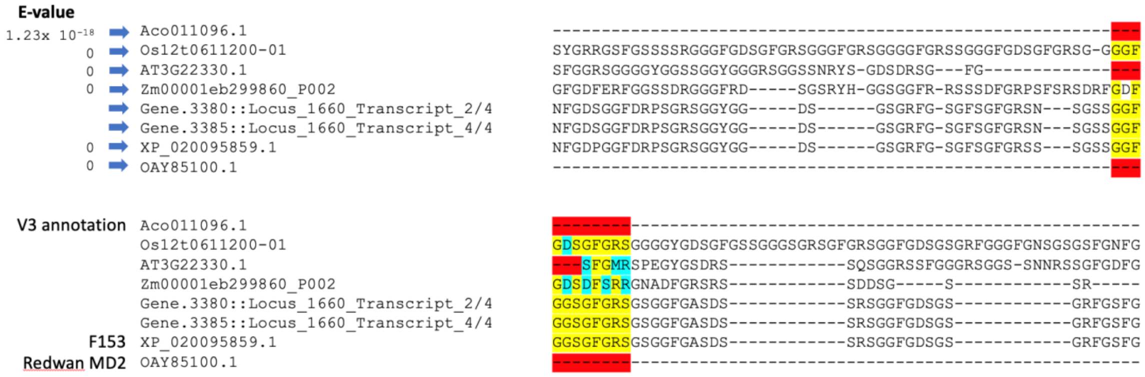

Figure S3. Multiple Sequence Alignment (MSA) of a potential novel paralog supported by novel peptides. The transcript "Locus_1660_Transcript" aligns to the V3 pineapple genome at an unannotated locus and returns a blast hit with a truncated sequence missing the region with the novel peptide. It matches with an MD2 protein that is similarly truncated and with a F153 protein that does include the region. Aligned MS-supported peptides are highlighted in yellow, with amino acid substitutions in the homologues shown in turquoise and missing sequence in red. BLASTX E-values for the Transcript versus each of the candidate proteins are shown left.

Figure S4. MSA of a potential novel paralog supported by novel peptides.

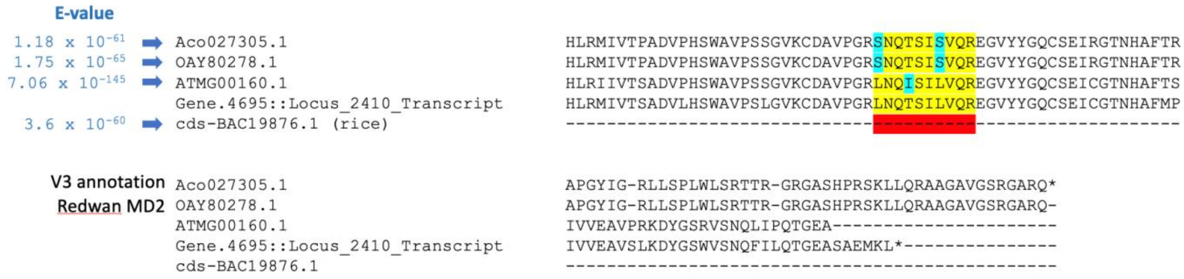

Figure S4. MSA of a potential novel paralog supported by novel peptides. The "Locus_2410_Transcript" does not align to the V3 genome but BLASTX returns matches to the proteins predicted from the V3 and MD2 pineapple genomes but not to the F153 proteome. Aligned MS-supported peptides are highlighted in yellow, with amino acid substitutions in the homologues shown in turquoise and missing sequence in red. BLASTX E-values for the transcript versus each of the candidate proteins are shown left.

Figure S5. MSA of Locus_7918_Transcript contig translation with plant protein homologues

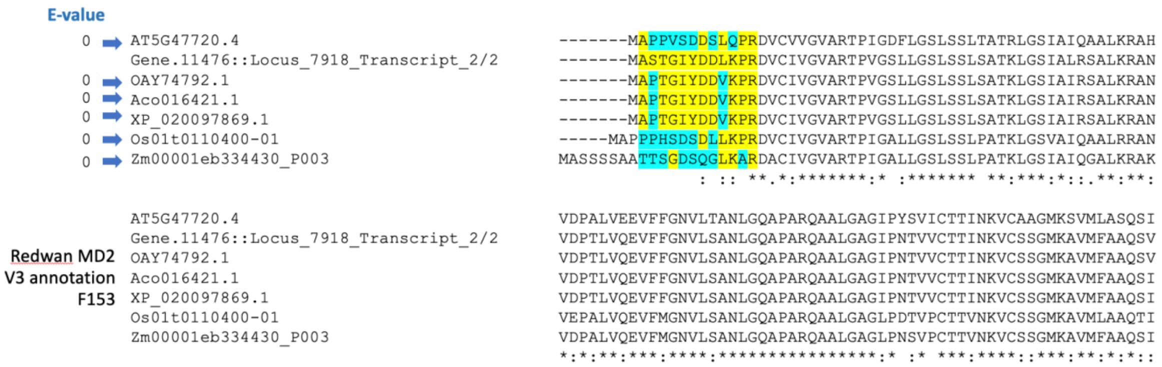

Figure S5. MSA of Locus_7918_Transcript contig translation with plant protein homologues. The transcript “Locus_7918_Transcript” aligns to the V3 genome at an unannotated locus, has two MS-detected peptides, and possesses homologues in 3 plant proteomes. The aligned MS-supported peptide region is highlighted in yellow, with amino acid substitutions in the homologues shown in turquoise and missing sequence in red. BLASTX E-values of the transcript versus each of the candidate proteins are shown left.
